# Supplementary material for: Heat adaptation of phage T7 under an extended genetic code
Source: Virus Evol. 2021 Dec 1;7(2):veab100. doi: 10.1093/ve/veab100 (PMC8923235; doi:10.1093/ve/veab100)
Supplement: veab100_Supp [file veab100_supp.zip › Sequencing_Analysis.zip!/Sequencing_Analysis/S1/evidence/JC_SIDE_1_58.html]

BRESEQ :: Evidence


| New junction evidence | | | | | | | | | | | |
| --- | --- | --- | --- | --- | --- | --- | --- | --- | --- | --- | --- |
|  | | seq id | position | reads (cov) | reads (cov) | score | skew | freq | annotation | gene | product |
| \* | ? | NC\_001604.1 | = 16508 | 1353 (0.320) | 2351 (0.570) | 369/586 | 0.2 | 62.6% | intergenic (–/–) | *–/–* | –/– |
|  | ? | NC\_001604.1 | 16970 = | 1483 (0.360) |  |  |  |  | intergenic (–/–) | *–/–* | –/– |

Only 100 of 3761 total aligned reads displayed.  

`CTCTCATCTGCAAACTGTGGATTATCAAGACCGAAGAGATGCTCGTAGAGAAAGGCTTGAAGCATGGCTGGGATGGGGACTTTGCGTACATGGCATGGGTACATGATGAAATCCAAGTAGGCTGCCGTACCGAAGAGATTGCTCAGGTGGTCATTGAGACCGCACAAGAAGCGATGCGCTGGGTTGGAGACCACTGGAACTTCCGGTGTCTTCTGGATACCGAAGGTAAGATGGGTCCTAATTGGGCGATTTGCCACTGATACAGGAGGCTACTCATGAACGAAAGACACTTAACAGGTGCTGCTTCTGAAATGCTAGTAGCCTACAAATTTACCAAAGCTGGGTACACTGTCTATTACCCTATGCTGACTCAGAGTAAAGAGGACTTGGTTGTATGTAAGGATGGTAAATTTAGTAAGGTTCAGGTTAAAACAGCCACAACGGTTCAAACCAACACAGGAGATGCCAAGCAGGTTAGGCTAGGTGGATGCGGTAGGTCCGAATATAAGGATGGAGACTTTGACATTCTTGCGGTTGTGGTTGACGAAGATGTGCTTATTTTCACATGGGACGAAGTAAAAGGTAAGACATCCATGTGTG  >  NC_001604.1/16208‑16807`  

                                                                                                                                                                                                                                                                                                            `|`                                                                                                                                                                                                                                                                                                             
    `cATCTGCAAACTGTGGATTATCAAGACCGAAGAGATGCTCGTAGAGAAAGGCTTGAAGCATGGCTGGGATGGGGACTTTGCGTACATGGCATGGGTACATGATGAAATCCAAGTAGGCTGCCGTACCGAAGAGATTGCTCAGGTGGTCATTGAGACCGCACAAGAAGCGATGCGCTGGGTTGGAGACCACAGGAACTTCCGGTGTCTTCTGGATACCGAAGGTAAGATGGGTCCTAATTGGGCGATTTGCCACTGATACAGGAGGCTACTCATGAACGAAAGACACTTAACAGGTGCgatt                                                                                                                                                                                                                                                                                                         <  1:27839‑M1/301‑5 (MQ=255)`  
     `aTCTGCAAACTGTGGATTATCAAGACCGAAGAGAAGCTCGTAGAGAAAGGCTTGAAGCATGGCTGGGATGGGGACTTTGCGTACATGGCATGGGTACATGATGAAATCCAAGTAGGCTGCCGTACCGAAGAGATTGCTCAGGTGGTCATTGAGACCGCAAAAGAAGCGATGCGCTGGGTTGGAGACCACTGGAACTTCCGGTGTCTTCTGGATACCGAAGGTAAGATGGGTCCTAATTGGGCGATTTGCCACTGATACAGGAGGCTACTCATGAACGAAAGACACTTAACAGGTGCgattg                                                                                                                                                                                                                                                                                                        <  1:313352‑M1/301‑6 (MQ=255)`  
      `tCTGCAAACTGTGGATTATCAAGACCGAAGAGATGCTCGTAGAGAAAGGCTTGAAGCATGGCTGGGATGGGGACTTTGCGTACATGGCATGGGTACATGATGAAATCCAAGTAGGCTGCCGTACCGAAGAGATTGCTCAGGTGGTCATTGAGACCGCACAAGAAGCGATGAGCTGGGTTGGAGACCACTGGAACTTCCGGTGTCTTCTGGATAACGAAGGTAAGATGGGTCCTAATTGGGCGCTTTGCCACTGATACAGGTGGCGACTCATGAAAGAAAGACACTTAACAGTGGCTGCTTc                                                                                                                                                                                                                                                                                                       >  1:377321/1‑301 (MQ=255)`  
          `tataagagacagtATCAAGAACGAAGAGATGCTCGTAGAGAAAGGCTTGAAGCATGGCTGGGATGGGGACTTTGCGTACATGGCATGGGTACATGATGAAATCCAAGTAGGCTGCCGTACCGAAGAGATTGCTCAGGTGGTCATTGAGACCGCACAAGAAGCGATGCGCTGGGTTGGAGACCACTGGAACTTCCGGTGTCTTCTGGATACCGAAGGTAAGATGGGTCCTAATTGGGCGATTTGCCACTGATACAGGAGGCTACTCATGAACGAAAGACACTTAACAGGTGCTGCTTCTGaa                                                                                                                                                                                                                                                                                                   <  1:198882/289‑1 (MQ=255)`  
          `cAAACTGAGGATTAACAAGAGCGAAGAGATGCTCGTAGAGAAAGGCTTGAAGGATGGCTGGGATGGGGACTTTGCGTACATGGCATGGGTACAGGATGAAATCCAAGTAGGCTGCCGTACCGAAGAGATTGCTCAGGTGGTCATTGAGACCGCACAAGAATCGATGTGCTGGGTTGGAGACCACTGGAACTTCCGGTGTCTTCTGGATACCGAAGGTAAGATGGGTCCTAATTGGGCGATTTGCCACTGATACAGGAGGCTACTCATGAACGAAAGACACTTAACAGGTGCgattgtcccc                                                                                                                                                                                                                                                                                                   <  1:597016‑M1/301‑11 (MQ=255)`  
              `cTGTGGATTATCAAGACCGAAGAGATGCTCGTAGAGAAAGGCTTGAAGCATGGCTGGGATGGGGACTTTGCGTACATGGCATGGGTACATGATGAAATCCAAGTAGGCTGCCGTACCGAAGAGATTGCTCAGGTGGTCATTGAGACCGCACAAGAAGCGATGCGCTGGGTTGGAGACCACTGGAACTTCCGGTGTCTTCTGGATACCGAAGGTAAGATGGGTCCTAATTGGGCGATTTGCCACTGATACAGGAGGCTACTCATGAACGAAAGACACTTAACAGGTGCTGCTTCTGAAATGc                                                                                                                                                                                                                                                                                               >  1:641759/1‑301 (MQ=255)`  
                     `ttATCAAGACCGAAGAGATGCTCGTAGAGAAAGGCTTGAAGCATGGCTGGGATGGGGACTTTGCGTACATGGCATGGGTACATGATGAAATCCAAGTAGGCTGCCGTACCGAAGAGATTGCTCAGGTGGTCATTGAGACCGCACAAGAAGCGATGCGCTGGGTTGGAGACCACTGGAACTTCCGGTGTCTTCTGGATACCGAAGGTAAGATGGGTCCTAATTGGGCGATTTGCCACTGATACAGGAGGCTACTCATGAACGAAAGACACTTAACAGGTGCTGCTTCTGAAATGCTAGTAGc                                                                                                                                                                                                                                                                                        >  1:333078/1‑301 (MQ=255)`  
                       `aTCAAGACCGAAGAGATGCTCGTAGAGAAAGGCTTGAAGCATGGCTGGGATGGGGACTTTGCGTACATGGCATGGGTACATGATGAAATCCAAGTAGGCTGCCGTACCGAAGAGATTGCTCAGGTGGTCATTGAGACCGCACAAGAAGCGATGCGCTGGGTTGGAGACCACTGGAACTTCCGGTGTCTTCTGGATACCGAAGGTAAGATGGGTCCTAATTGGGCGATTTGCCACTGATACAGGAGGCTACTCATGAACGAAAGACACTTAACAGGTGCgattgtccccaatggtaaacaga                                                                                                                                                                                                                                                                                      >  1:298482‑M1/1‑278 (MQ=255)`  
                       `aTCAAGACCGAAGAGATGCTCGTAGAGAAAGGCTTGAAGCATGGCTGGGATGGGGACTTTGCGTACATGGCATGGGCACATGATGAAATCCAAGTAGGCTGCCGTACCGAAGAGATTGCTCAGGTGGTCATTGAGACCGCACAAGAAGCGATGCGCTGGGTTGGAGACCACTGGAACTTCCGGTGTCTTCTGGATACCGAAGGTAAGATGGGTCCTAATTGGGCGATTTGCCACTGATACAGGAGGCTACTCATGAACGAAAGACACTTAACAGGTGCgattgtccccaatggtaaacaga                                                                                                                                                                                                                                                                                      >  1:16207‑M1/1‑278 (MQ=255)`  
                       `aTCAAGACCGAAGAGATGCTCGTAGAGAAAGGACTGCAGCATGGCTGGGATGGCAACTTTGCGTACATTGCCAGGGTACATGATGAAATCCAAGTAGGCAGCAGTACCGAAGAGATTGCTCAGGTGGTCATTGAGCCCGCACAACAAGCGATGCGCTAGGTTGGAGACAACTGGAACTTCTGGTGTCTTCGGGATAACGAAGGTAAGAAGAGTCATAAGTGGGCGATTTGCCACTGATACAGGAGGATAATAATGAACGAAAGAGAATAAACAGGagcgattgtccccgacggtacacaca                                                                                                                                                                                                                                                                                      >  1:352972/1‑278 (MQ=255)`  
                         `aaagAGCGAAGGGATGCTCGTAGAGAAAGGCTTGAAGAATGGCTGGGATGGGGAGTTTGCGTACATGGCATGGGTACATTATGAAATAAAAGTAGGCTGCCGTACAGAAGAGAGTGCGCAGGTGGTCATTGAGACCGCACAAGAAGCGATGCGCTGGGTTGGAGACCAATGGAACTTCCGGTGTCTTCTGGATACCGAAGGTAAGATGGGTACTAATTGGGCGATTTGCAAATGATAAAGGAGGCTACTAATGAACGAAAGACACTTAACAGGTGCgattgtccccaatggtaaacagaag                                                                                                                                                                                                                                                                                    <  1:364246‑M1/300‑26 (MQ=255)`  
                             `aCCGAAGAGATGCTCGTAGAGAAAGGCTTGAAGCATGGCTGGGATGGGGACTTTGCGTACATGGCATGGGTACACGATGAAATCCAAGTAGGCTGCCGTACCGAAGAGATTGCTCAGGTGGTCATTGAGACCGCACAAGAAGCGATGCGCTGGGTTGGAGACCACTGGAACTTCCGGTGTCTTCTGGATACCGAAGGTAAGATGGGTCCTAATTGGGCGATTTGCCACTGATACAGGAGGCTACTCATGAACGAAAGACACTTAACAGGTGCTGCTTCTGCAATGCTAGTAGCCTAAAAAt                                                                                                                                                                                                                                                                                >  1:348047/1‑301 (MQ=255)`  
                                `gAAGAGATGTGCGTAGAGAAAGGCTTGAAGCATGGCGGGGATGGGGACTTTGCGTACATGGCATGGGTACATGATGAAATCCAAGTAGGCTGCCGTACCGAAGAGATTGCTCAGGTGGTCATTGAGACCGCACAAGAAGCGATGCGCTGGGTTGGAGACCACTGGAACTTCCGGTGTCTTCTGGATACCGAAGGTAAGATGGGTCCTAATTGGGCGATTTGCCACTGATACAGGAGGCTACTCATGAACGAAAGACACTTAACAGGTGCgattgtccccaatggtaaacagaaggaaatga                                                                                                                                                                                                                                                                             <  1:358436‑M1/301‑33 (MQ=255)`  
                                     `gaTGCTCGAAGAGAAAGGCTTGAAGCATGGCTGGGATGGGGACTTTGCGTACATGGCATGGGTACATGATGAAATCCAAGTAGGCTGCCGTACCGAAGAGATTGCTCAGGTGGTCATTGAGACCGCACAAGAAGCGATGCGCTGGGTTGGAGACCACTGGAACTTCCGGTGTCTTCTGGATACCGAAGGTAAGATGGGTCCTAATTGGGCGATTTGCCACTGATACAGGAGGCTACTCATGAACGAAAGACACTTAACAGGTGCTGCTTCTGAAATGCTAGTAGCCTACAAATTTACCaaa                                                                                                                                                                                                                                                                        >  1:628443/1‑301 (MQ=255)`  
                                           `ggtAGAGAAAGGCTTGAAGCAGGGCTGGGATGGGGACTTTGCGTACGTGGCATGGGTACATGATGAAATCCAAGTAGGCTGCCGTACCGAAGAGATTGCTCAGGTGGTCATTGAGACCGCACAAGAAGCGATGCGCTGGGTTGGAGACCACTGGAACTTCCGGTGTCTTCTGGATACCGAAGGTAAGATGGGTCCTAATTGGGCGATTTGCCACTGATACAGGAGGCTACTCATGAACGAAAGACACTTAACAGGTGCgattgtccccaatggtaaacagaaggaaatgattgtccagttc                                                                                                                                                                                                                                                                  <  1:571396‑M1/300‑44 (MQ=255)`  
                                           `cGTAGAGAAAGGCTTGAAGCATGGCTGGGATGGGGACTTTGCGTACATGGCATGGGTACATAATGAAATCCAAGTAGGCTGCCGTACCGAAGAGATTGCTCAGGTGGTCATTGAGACCGCACAAGAAGCGATGCGCTGGGTTGGAGACCACTGGAACTTCCGGTGTCTTCTGGATACCGAAGGTAAGATGGGTCCTAATTGGGCGATTTGCCACTGATACAGGATGCTACTCATGAACGAAAGACACTTAACAGGTGCgattgtccccaatggtaaacagaaggaaatgattgtccagttc                                                                                                                                                                                                                                                                  <  1:411210‑M1/301‑44 (MQ=255)`  
                                            `acagagaaAGGCTTGAAGCATGGCTGGGATGGGGACTTTGCGTACATGGCATGGGTACATTATGAAATCCAAGTAGGCTGCCGTACCGAAGAGATTGCTCAGGTGGTCATTGAGACCGCACAAGAAGCGATGCGCTGGGTTGGAGACCACTGGAACTTCCGGTGTCTTCTGGATACCGAAGGTAAGATGGGTCCTAATTGGGCGATTTGCCACTGATACAGGAGGCTACTCATGAACGAAAGACACTTAACAGGTGCgattgtccccaatggtaaacagaaggaaatgattgtccagttcc                                                                                                                                                                                                                                                                 <  1:436947‑M1/299‑45 (MQ=255)`  
                                              `agagAAAGGCTTGAAGCATGGCTGGGATGGGGACTTTGCGTACATGGCATGGGTACATGATGAAATCCAAGTAGGCTGCCGTACCGAAGAGATTGCTCAGGTGGTCATTGAGACCGCACAAGAAGCGATGCGCTGGGTTGGAGACCACTGGAACTTCCGGTGTCTTCTGGATACCGAAGGTAAGATGGGTCCTAATTGGGCGATTTGCCACTGATACAGGAGGCTACTCATGAACGAAAGACACTTAACAGGTGCTGCTTCTGAAATGCTAGTAGCCTACAAATTTACCAAAGCTGGGTac                                                                                                                                                                                                                                                               <  1:226167/301‑1 (MQ=255)`  
                                                `agaAAGGCTTGAAGCATGGCTGGGATGGGGACTTTGCGTACATGGCATGGGTACATGATGAAAGCCAAGTAGGCTGCCGTACCGAAGAGAGTGCTCAGGTGGTCATTGAGACCGCACAAGAAGCGATGCGCTGGGTTGGAGACCACTGGAACTTCCGGTGTCTGCTGGATACCGAAGGTAAGATGGGTCCTAATTGGGCGATTTGCCACTGATACAGGAGGCTACTCATGAACGAAAGACACTTAACAGGTGCgattgtccccaatggtaaacagaaggaaatgattgtccagttcctgac                                                                                                                                                                                                                                                             <  1:285634‑M1/301‑49 (MQ=255)`  
                                                     `ggCTTGAAGCATGGCTGGGATGGGGACTTTGCGTACATGGCATGGGTACATGATGAAATCCAAGTAGGCTGCCGTACCGAAGAGATTGCTCAGGTGGTCATTGAGACCGCACAAGAAGCGATGCGCTGGGTTGGAGACCACTGGAACTTCCGGTGTCTTCTGGATACCGAAGGTAAGATGGGTCCTAATTGGGCGATTTGCCACTGATACAGGAGGCTACTCATGAACGAAAGACACTTAACAGGTGCTGCTTCTGAAATGCTAGTAGCCTACAAATTTACCAAAGCTGGGTACACTGTCt                                                                                                                                                                                                                                                        >  1:305302/1‑301 (MQ=255)`  
                                                          `gAAGCATGGCTGGGATGGGGACTTTGCGTACATGGAATGGGTACATGATGAAATCCAAGTAGGCTGCCGTACCGAAGAGATTGCTCAGGTGGTCATTGAGACCGCACAAGAAGCGATGCGCTCGGTTGGAGACCACTGGAACTTCCGGTGTCTTCTGGATACCGAAGGTAAGATGGGTCCTAATTGGGCGATTTGCCACTGATACAGGAGGCTACTCATTAACGAAAGACACTTAACAGGTGCTGCTTCTGAAATGCTAGTAGCCTACAAATTTACCAAAGCTGGGTACACTGTCTATTAc                                                                                                                                                                                                                                                   <  1:53138/301‑1 (MQ=255)`  
                                                            `aGCATGGCTGGGATGGGGACTTTGCGTACATGGCATGGGTACATGATGAAATCCAAGTAGGCTGCCGTACCGAAGAGATTGCTCAGGTGGTCATTGAGACCGCACAAGAAGCGATGCGCTGGGTTGGAGACCACTGGAACTTCCGGTGTCTTCTGGATACCGAAGGTAAGATGGGTCCTAATTGGGCGATTTGCCACTGATACAGGAGGCTACTCATGAACGAAAGACACTTAACAGGTGCTGCTTCTGAAATGCTAGTAGCCTACAAATTTACCAAAGCTGGGTACACTGTCTATTAccc                                                                                                                                                                                                                                                 >  1:590274/1‑301 (MQ=255)`  
                                                             `gCATGGCTGGGATGGGGACTTTGCGTACATGGCATGGGTACATGATGAAATCCAAGTAGGCTGCCGTACCGAAGAGATTGCTCAGGTGGTCATTGAGACCGCACAAGAAGCGATGCGCTGGGTTGGAGACCACTGGAACTTCCGGTGTCTTCTGGATACCGAAGGTAAGATGGGTCCTAATTGGGCGATTTGCCACTGATACAGGAGGCTACTCATGAACGAAAGACACTTAACAGGTGCgattgtccccaatggtaaacagaaggaaatgattgtccagttcctgacacacggtatggaa                                                                                                                                                                                                                                                >  1:75119‑M1/1‑240 (MQ=255)`  
                                                              `cATGGCGGGGATGGGGACTTTGCGTACATGGCATGGGTACATGATGAAATCCAAGTAGGCTGCCGTACCGAAGAGATTGCTCAGGTGGTCATTGAGACCGCACAAGAAGCGATGCGCTGGGTTGGAGACCACTGGAACTTCCGGTGTCTTCTGGATACCGAAGGTAAGATGGGTCCTAATTGGGCGATTTGCCACTGATACAGGAGGCTACTCATGAACGAAAGACACTTAACAGGTGCgattgtccccaatggtaaacagaaggaaatgattgtccagttcctgacacacggtatggaag                                                                                                                                                                                                                                               <  1:319882‑M1/301‑63 (MQ=255)`  
                                                               `aTGGCTGGTATGGTGACTTTGCGTACATGGCATGGGTACATGATGAAAGCCAAGTAGGCTGCCGGACCGAAGAGATTGCTCAGGTGGTCATTGAGCCCGCACAAGAAGCGATGCGCTGGGTTGGAGACCACTGGAACTTCCGGTGTCTTCTGGATACCGAAGGTAAGATGGGTCCTAATTGGGCGATTTGCCACTGATACAGGAGGCTACTCATGAACGAAAGACACTTAACAGGTGCgattgtccccaatggtaaacaggaggaaatgattgtccagttcctgacacacggtatggaagg                                                                                                                                                                                                                                              <  1:118020‑M1/301‑64 (MQ=255)`  
                                                                `tGGCTGGGATGGGGACTTTGCGTACATGGCATGGGTACATGATGAAATCCAAGTAGGCTGCCGTACCGAAGAGATTGCTCAGGTGGTCATTGAGACCGCACAAGAAGCGATGCGCTGGGTTGGAGACCACTGGAACTTCCGGTGTCTTCTGGATACCGAAGGTAAGATGGGTCCTAATTGGGCGATTTGCCACTGATACAGGAGGCTACTCATGAACGAAAGACACTTAACAGGTGCgattgtccccaatggtaaacagaaggaaatgattgtccagttcctgacacacggtatggaagga                                                                                                                                                                                                                                             >  1:212436‑M1/1‑237 (MQ=255)`  
                                                                `tGGCTGGGATGGGGACTTTGCGTACATGGCATGGGTACATGATGAAATCCAAGTAGGCTGCCGTACCGAAGAGATTGCTCAGGTGGTCATTGAGACCGCACAAGAAGCGATGCGCTGGGTTGGAGACCACTGGAACTTCCGGTGTCTTCTGGATACCGAAGGTAAGATGGGTCCTAATTGGGCGATTTGCCACTGATACAGGAGGCTACTCATGAACGAAAGACACTTAACAGGTGCgattgtccccaatggtaaacagaaggaaatgattgtccagttcctgacacacggtatggaagga                                                                                                                                                                                                                                             >  1:254512‑M1/1‑237 (MQ=255)`  
                                                                 `ggCTGGGATGGGGACTTTGCGTACCTGGCATGGGTACATGATGAAATCCAAGTAGGCTGCCGTACCGAAGAGATTGCTCAGGTGGTCATTGAGACCGCACAAGAAGCGATGCGCTGGGTTGGAGACCACTGGAACTTCCGGTGTCTTCTGGATACCGAAGGTAAGATGGGTCCTAATTGGGCGATTTGCCACTGATACAGGAGGCTACTCATGAACGAAAGACACTTAACAGGTGCTGCTTCTGAAATGCTAGTAGCCTACAAATTTACCAAAGCTGGGTACACTGTCTATTACCCTATGc                                                                                                                                                                                                                                            <  1:515781/301‑1 (MQ=255)`  
                                                                 `ggCTGGGATGGGGACTTTGCGTACATGGCATGTGTACATGATGAAATCAAAGTAGGCTGCCGTACCGAAGAGATTGATCAGGTAGTAATTGAGACCGCACAAGAAGCGATGCGCTGGGTTGGAGACCACTGGAACTTCCGGTGTCTTCTGGATACCGAAGGTAAGATGGGTCCTAATTGAGCGATTTGCCACTGATACAGGAGGCTACTCATGAACGAAAGACACTTAACAGGTGCTGCTTCTGAAATGCTAGTAGCTTACAAATTTACCAAAGCTGGGTACACTGTCTATTACCCTATGc                                                                                                                                                                                                                                            <  1:272565/301‑1 (MQ=255)`  
                                                                 `ggCTGGGATGGGGACTTTGCGTACATGGCATGGGTACATGATGAAATCCAAGTAGGCTGCCGTACCGAAGAGATTGCTCAGGTGGTCATTGAGACCGCACAAGAAGCGATGCGCTGGGTTGGAGACCACTGGAACTTCCGGTGTCTTCTGGATACCGAAGGTAAGATGGGTCCTAATTGTGCGAATTGCCACTGATACAGTATGCTACTCATGAACGAAAGACACTTAACAGGTGCgattgtccccaatggtaaacagaaggaaatgattgtccagttccttacacacgttatggaaggtt                                                                                                                                                                                                                                            >  1:509047‑M1/1‑236 (MQ=255)`  
                                                                  `gCTGGGATGGGGACTTTGCGTACATGGCATGGGTACATGATGAAATCCAAGTAGGCTGCCGTACCGAAGAGATTGCTCAGGTGGTCATTGAGACCGCACAAGAAGCGATGCGCTGGGTTGGAGACCACTGGAACTTCCGGTGTCTTCTGGATACCGAAGGTAAGATGGGTCCTAATTGGGCGATTTGCCACTGATACAGGAGGCTACTCATGAACGAAAGACACTTAACAGGTGCgattgtccccaatggtaaacagaatgaaatgattgtccagttcctgacacacggtatggaaggatt                                                                                                                                                                                                                                           >  1:70326‑M1/1‑235 (MQ=255)`  
                                                                       `gATGGGGACTTTGCGTACATGGCATGGGTACATGATGAAATCCAAGTAGGCTGCCGTACCGAAGAGATTGCTCAGGTGGTCATTGAGACCGCACAAGAAGCGATGCGCTGGGTTGGAGACCACTGGAACTTCCGGTGTCTTCTGGATACCGAAGGTAAGATGGGTCCTAATTGGGCGATTTGCCACTGATACAGGAGGCTACTCATGAACGAAAGACACTTAACAGGTGCTGCTTCTGAAATGCTAGTAGCCTGCAAATTTACCAAAGCTGGGTACACTGTCTATTAcccctgtctcttat                                                                                                                                                                                                                                      >  1:325484/1‑290 (MQ=255)`  
                                                                               `cTTTGCGTACATGGCATTGGTACATGATGAAATCCAAGTAGACTGCCGTACCGAAGAGATTGCTCAGGTGGTCATTGAGACCGCACAAGAAGCGATGCGCTGGGTTGGAGACCTCTGGAACTTCCGGTGTCTTCTGGATACCGAAGGTAAGATGGGTCCTAATTGGGCGATTTGCCACTGATGCAGGAGGCTACTCATGAACGAAAGACACTTAACAGGTGCgattgtccccaatggtaaacagaaggaaatgattgtccagttcctgacacacggtatggaaggattgatgacattcgta                                                                                                                                                                                                                              >  1:87519‑M1/1‑222 (MQ=255)`  
                                                                               `cTTTGCGTACATGGCATGGGTACATGATGAAATCCAAGTAGGCTGCCGTACCGAAGAGATTGCCCAGGTGGTCATTGAGACCGCACAAGAAGCGATGCGCTGGGTTGGAGACCACTGGAACTTCCGGTGTCTTCTGGATACCGAAGGTAAGATGGGTCCTAATTGGGCGATTTGCCACTGATACAGGAGGCTACTCATGAACGAAATACACTTAACAGGTGCgattgtccccaatggtaaacagaaggaaatgattgtccagttcctgacacacggtatggaaggattgatgacattcgtt                                                                                                                                                                                                                              >  1:417815‑M1/1‑222 (MQ=255)`  
                                                                                   `ggagatgtgtataagagaCAGGATGAAATCCAAGTAGGCTGCCGTACCGAAGAGATTGCTCAGGTGGTCATTGAGACCGCACAAGAAGCGATGCGCTGGGTTGGAGACCACTGGAACTTCCGGTGTCTTCTGGATACCGAAGGTAAGATGGGTCCTAATTGGGCGATTTGCCACTGATACAGGAGGCTACTCATGAACGAAAGACACTTAACAGGTGCTGCTTCTGAAATGCTAGTAGCCTACAAATTTACCAAAGCTGGGTACACTGTCTATTACCCTATGCTGACTCAGAGTAAAGAgg                                                                                                                                                                                                                          <  1:5851/284‑1 (MQ=255)`  
                                                                                               `tggGTACATGATGAAATCCAAGTAGGCTGCCGTACCGAAGAGATTGCTCAGGTGGTCATTGAGACCGCACAAGAAGCGATGCGCTGGGTTGGAGACCACTGGAACTTCCGGTGTCTTCTGGATACCGAAGGTAAGATGGGTCCTAATTGGGCGATTTGCCACTGATACAGGAGGCTACTCATGAACGAAAGACACTTAACAGGTGCgattgtccccaatggtaaacagaaggaaatgattgtccagttcctgacacacggtatggaaggattgatgacattcgtagtacgtacatcatttc                                                                                                                                                                                                              >  1:337846‑M1/1‑206 (MQ=255)`  
                                                                                                `gggTACATGATGAAATCCAAGTAGGCTGCCGTACCGAAGAGATTGCTCAGGTGGTCATTGAGACCGCACAAGAAGCGATGCGCTGGGTTGGAGACCACTGGAACTTCCGGTGTCTTCTGGATACCGAAGGTAAGATGGGTCCTAATTGGGCGATTTGCCACTGATACAGGAGGCTACTCATGAACGAAAGACACTTAACAGGTGCTGCTTCTGAAATGCTAGTAGCCTACAAATTTACCAAAGCTGGGTACACTGTCTATTACCCTATGCTGACTCAGAGTAAagactgtctcttatacac                                                                                                                                                                                                             >  1:536552/1‑286 (MQ=255)`  
                                                                                                  `tataagtgacagatcgaaggagggtGACGTACCGAAGAAATTGCTCAGGGGGTCATTGAGACAGCACAAGAAGCGATGGGCTGGGTTGGAGACCACTGGAACTGCCGGTGTCTTCTGGATACCGAAGGTAAGATGGGTACTAATTGGGCGATTTGCCACTGATACAGGAGGCTACTCATGAACGAAAGACACTTAACAGGTGCgattgtccccaatggtaaacagaaggaaatgattgtccagttcctgacacacggtatggaaggattgatgacattcgtagtacgtacatcatttcgtg                                                                                                                                                                                                           <  1:469516‑M1/289‑99 (MQ=255)`  
                                                                                                  `gTACATGATGAAATCCAAGTAGGCTGCCGTACCGAAGAGATTGCTCAGGTGGTCATTGAGACCGCACAAGAAGCGATGCGCTGGGTTGGAGACCACTGGAACTTCCGGTGTCTTCTGGATACCGAAGGTAAGATGGGTCCTAATTGGGCGATTTGCCACTGATACAGGAGGCTACTCATGAACGAAAGACACTTAACAGGTGCTGCTTCTGAAATGCTAGTAGCCTACAAATTTACCAAAGCTGGGTACACTGTCTATTACCCTATGCTGACTCAGAGTAAAGAGGACTTGGTtgtatgta                                                                                                                                                                                                           >  1:634756/1‑301 (MQ=255)`  
                                                                                                    `aCATGATGAAATCCAAGTAGGCTGCCGTACCGAAGAGATTGCTCAGGTGGTCATTGAGACCGCACAAGAAGCGTTGAGCTGGGTTGGAGACCACTGGAACTTCCAGTGTCTTCTGGATACCGAAGGTAAGATGGGTCCTCATTGGGCGATTTGCCCCTGATACAGGAGCCTACTCATTAACGAAAGCCACTTAACAGCTGCgattgtacccaatggtaaccagaaggagataattgtcctgttcctgacactcggtatgaaaggattgatgtcattcgtgttaacatctctactacacttt                                                                                                                                                                                                         >  1:578627‑M1/1‑201 (MQ=255)`  
                                                                                                       `tgatgaAATCCAAGTAGGCTGCCGTACCGAAGAGATTGCTCAGGTGGTCATTGAGACCGCACAAGAAGCGATGCGCTGGGTTGGAGACCACTGGAACTTCCGGTGTCTTCTGGATACCGAAGGTAAGATGGGTCCTAATTGGGCGATTTGCCACTGATACAGGAGGCTACTCATGAACGAAAGACACTTAACAGGTGCgattgtccccaatggtaaacagaaggaaatgattgtccagttcctgacacacggtatggaaggattgatgacattcgtagtacgtacatcatttcgtgaggcc                                                                                                                                                                                                      >  1:268121‑M1/1‑198 (MQ=255)`  
                                                                                                        `gatgaAATCCAAGTAGGCTGCCGTACCGAAGAGATTGCTCAGGTGGTCATTGAGACCGCACAAGAAGCGATGCGCTGGGTTGGAGACCACTGGAACTTCCGGTGTCTTCTGGATACCGAAGGTAAGATGGGTCCTAATTGGGCGATTTGCCACTGATACAGGAGGCTACTCATGAACGAAAGACACTTAACAGGTGCgattgtccccaatggtaaacagaaggaaatgattgtccagttcctgacacacggtatggaaggattgatgacattcgtagtacgtacatcatttcgtgaggcca                                                                                                                                                                                                     >  1:24329‑M1/1‑197 (MQ=255)`  
                                                                                                               `tCCAAGTAGGCTGCCGTACCGAAGAGATTGCTCAGGTGGTCATTGAGACCGAACAAGAAGCGATGCGCTGGGTTGGAGACCACTGGAACTTCCGGTGTCTTCTGGATACCGAAGGTAAGATGGGTCCTAATTGGGCGATTTGCCACTGATACAGGAGGCTACTCATGAACGAAAGACACTTAACAGGTGCgattgtccccaatggtaaacagaaggaaatgattgtccagttcctgacacacggtatggaaggattgatgacattcgtagtacgtacatcatttcgtgaggccattaagga                                                                                                                                                                                              <  1:400391‑M1/301‑112 (MQ=255)`  
                                                                                                                 `cAAGTAGGCTGCCGTACCGAAGAGATTGCTCAGGTGGTCATTGAGACCGCACAAGAAGCGATGCGCTGGGTTGGAGACCACTGGAACTTCCGGTGTCTTCTGGATACCGAAGGTAAGATGGGTCCTAATTGGGCGATTTGCCACTGATACAGGAGTCTACTCATGAACGAAAGACACTTACCAGGTGCgattttccccaatggtaaacagaaggaaatgattttccagttcctgacacacggtatggaaggattgacgacattctttgtccttacatcatctgcctcttttacacatttcc                                                                                                                                                                                            >  1:655341‑M1/1‑188 (MQ=255)`  
                                                                                                                    `gTAGGCTGCCGTACCGAAGAGATTGCTCAGGTGGTCATTGAGACCGCACAAGAAGCGATGCGCTGGGTTGGAGACCACTGGAACTTCCGGTGTCTTCTGGATACCGAAGGTAAGATGGGTCCTAATTGGGCGATTTGCCACTGATACAGGAGGCTACTCATGAACGAAAGACACTTAACAGGTGCTGCTTCTGAAATGCTAGTAGCCTACAAATTTACCAAAGCTGGGTACACTGTCTATTACCCTATGCTGACTCAGAGTAAAGAGGACTTGGTTGTATGTAAGGATGGTAAATTTAGTa                                                                                                                                                                                         >  1:394683/1‑301 (MQ=255)`  
                                                                                                                          `ggcCGTACCGAAGAGATTGCTCAGGTGGTCATTGAGACCGCACAAGAAGCGATGCGCTGGGTTGGAGACCACTGGAACTTCCGGTGTCTTCTGGATACCGAAGGTAAGATGGGTCCTAATTGGGCGATTTGCCACTGATACAGGAGGCTACTCATGAACGAAAGACACTTAACAGGTGCgattgtccccaatggtaaacagaaggaaatgattgtccagttcctgacacacggtatggaaggattgatgacattcgtagtacgtacatcatttcgtgaggccattaaggacatgcacgaag                                                                                                                                                                                   <  1:237028‑M1/300‑123 (MQ=255)`  
                                                                                                                          `accactatcgaagagatTGCTAGGGTGGTCATTGAGAAAGCAGAAGAAGCGATGGGGTGGGTTAGAGACACGTGGAACTTACGGTGTCTTCTGGATACCGAAGGTAAGAGGGGACCTAGTTGGGCCATTGGCCGCTGATGCAGGAGGCTACTCATGAACGAAGGACACATAAGAGATGCgatggtactcaatggtaaacagaaggaaatgattgtccagttcctgacactcggtatggaaggattgatgacattcgtagtacgtacatcatttcgtgaggccattaaggacatgcacgaag                                                                                                                                                                                   <  1:521480‑M1/293‑123 (MQ=255)`  
                                                                                                                              `gTACCGAAGAGATTGCTCAGGTGGTCATTGAGACCGCACAAGAAGCGATGCGCTGGGTTGGAGACCACTGGAACTTCCGGTGTCTTCTGGATACCGAAGGTAAGATGGGTCCTAATTGGGCGATTTGCCACTGATACAGGAGGCTACTCATGAACGAAAGACACTTAACAGGTGCgattgtccccaatggtaaacagaaggaaatgattgtccagttcctgacacacggtatggaaggattgatgacattcgtcgtacgtacatcatttcgtgaggccattaaggacatgcacgaagagta                                                                                                                                                                               >  1:249081‑M1/1‑175 (MQ=255)`  
                                                                                                                                 `ccGGAGGGATTGCTCAGGTGGTAATTGAGTACGGACAAGAAGGGATGCTCTGGGTTGTAGACCACTGGAACTACCGGTGTCTTCTGGATACCGAAGGTAAGATGGGTCCTAATTGGGCGATTTGCCAGTGATACAGGAGGCTACTCATGAACGAATGAAACTTAACAGGTGCgattgtccccaatggtaaacagaaggaaatgattgtccagttcctgacacacggtatggaaggattgatgacattcgtggtacgtacatcatttcgtgaggccattaaggacatgcacgaagagtatgc                                                                                                                                                                            <  1:543563‑M1/301‑130 (MQ=255)`  
                                                                                                                                 `ccGAAGAGATTGCTCAGGTGGTCATTGAGACCGCACAAGAAGCGATGCGCTGGGTTGGAGACCACTGGAACTTCCGGTGTCTTCTGGATACCGAAGGTAAGATGGGTCCTAATTGGGCGATTTGCCACTGATACAGGAGGCTACTCATGAACGAAAGACACTTAACAGGTGCTGCTTCTGAAATGCTAGTAGCCTACAAATTTACCAAAGCTGGGTACACTGTCTATTACCCTATGCTGACTCAGAGTAAAGAGGACTTGGTTGTATGTAAGGATGGTAAATTTAGTAAGGTTCAGGTTaa                                                                                                                                                                            >  1:424768/1‑301 (MQ=255)`  
                                                                                                                                    `atgtgtataagagacaggtCATTGAGACCGCACAAGAAGCGATGCGCTGGGTTGGAGACCACTGGAACTTCCGGTGTCTTCTGGATACCGAAGGTAAGATGGGTCCTAATTGGGCGATTTGCCACTGATACAGGAGGCTACTCATGAACGAAAGACACTTAACAGGTGCTGCTTCTGAAATGCTAGTAGCCTACAAATTTACCAAAGCTGGGTACACTGTCTATTACCCTATGCTGACTCAGAGTAAAGAGGACTTGGTTGTATGTAAGGATGGTAAATTTAGTAAGGTTCAGGTTAAAAc                                                                                                                                                                         <  1:167011/285‑1 (MQ=255)`  
                                                                                                                                    `aaGAGATTGCTCAGGTGGTCATTGAGGCCGCACAAGAAGCGATGCGCTGGGTTGGAGACCACTGGAACTTCCGGTGTCTTCTGGATACCGAAGGTAATATGGGTCCTAATTGGGCGATTTGCCACTGATACAGGAGGCTACTCATGAACGAAAGACACTTAACAGGTGCgattgtccccaatggtaaacagaaggaaatgattgtccagttcctgacacacggtatggaaggattgatgacattcgtagtacgtacatcatttcgtgaggccattaaggacatgcacgaagagtatgcaga                                                                                                                                                                         <  1:463254‑M1/301‑133 (MQ=255)`  
                                                                                                                                    `aaGAGATTGCTCAGGTGGTCATTGAGACCGCACAAGAAGCGATGCGCTGGGTGGGAGACCACTGGAACTTCCGGTGTCTTCTGGATACCGAAGGTAAGATGGGTCCTAATTGGGCGATTTGCCACTGATACAGGAGGCTACTCATGAACGAAAGACACTTAACAGGTGCgattgtccccaatggtaaacagaaggaaatgattgtccagttcctgacacacggtatggaaggattgatgacattcgtagtacgtacatcatttcgtgaggccattaaggacatgcacgaagagtatgcaga                                                                                                                                                                         <  1:44771‑M1/301‑133 (MQ=255)`  
                                                                                                                                     `agagGTTGCTCAGGTGTGCATTGAGACCGCACAAGGAGCGATGCGCTGGGTTGGAGACCACTGGAACTTCCGGTGTCTTCTGGATACCGAAGGTAAGATGGGTCCTAATTGGGCGATTTGCCACTGATACATGAGGCTACTCATGAACGAAAGACACTTAACATGTGCgattttccccaatggtaaacagaaggaaatgtttgtccagttcctgacacacggtatggaaggattgatgacattcgtagtacgtacatcatttcgtgaggccattaaggacatgcacgaagagtatgcagat                                                                                                                                                                        <  1:173422‑M1/301‑134 (MQ=255)`  
                                                                                                                                         `atggctcAGGTTGTCATTGAGACCGAACAAGAAGCGATGCGCTGGGTTGGAGACCACTGGAACTTCCGGTGTCTTATGGATACCGAAGGTAAGATGGGTCCTAATTGGGCGATTTGCCACTGATACAGGAGGCTACTCATGAACGAAAGACACTTAACAGGTGCgattgtccccaatggtaaacagaaggaaatgattgtccagttcctgacacacggtatggaaggattgatgacattcgtagtacgtacatcatttcgtgaggccattaaggacatgcacgaagagtatgcagataagg                                                                                                                                                                    <  1:383091‑M1/298‑138 (MQ=255)`  
                                                                                                                                         `aTTGCTCAGGTGGTCATTGAGACCGCACAAGAAGCGATGCGCTGGGTTGGAGACCACTGGAACTTCCGGTGTCTTCTGGATACCGAAGGTAAGATGGGTCCTAATTGGGCGATTTGCCACTGATACAGGAGGCTACTCATGAACGAAAGACACTTAACAGGTGCgattgtccccaatggtaaacagaaggaaatgattgtccagttcctgacacacggtatggaaggattgatgacattcgtagtacgtacatcatttcgtgaggccattaaggacatgcacgaagagtatgcagataagg                                                                                                                                                                    >  1:388805‑M1/1‑164 (MQ=255)`  
                                                                                                                                          `ttGCTCAGGTGGTCATTGAGACCGCACAAGAAGCGATGCGCTGGGTTGGAGACCACTGGAACTTCCGGTGTCTTCTGGATACCGAAGGTAAGATGGGTCCTAATTGGGCGATTTGCCACTGATACAGGAGGCTACTCATGAACGAAAGACACTTAACAGGTGCgattgtccccaatggtaaacagaaggaaatgattgtccagttcctgacacacggtatggaaggattgatgacattcgtagtacgtacatcatttcgtgaggccattaaggacatgcacgaagagtatgcagactgtct                                                                                                                                                                   >  1:132783‑M1/1‑163 (MQ=255)`  
                                                                                                                                             `cTCAAGTTGTCATTGAGACCGCACAAGGAGCGATGCGCTTGGTTGGAGACCACTGGAACTTCCGGTGTCTTCTGGATACCGAAGGTAAGATGGGTCCTAATTTGGCGATTTGCCACTGATACAGGAGGCTACTCATGAACGAAAGACACTTAACAGGTGCgattgtccccaatggtaaacagaaggaaatgattgtccagttcctgacacacggtatggaaggattgatgacattcgtagtacgtacatcatttcgtgaggccattaaggacatgcacgaagagtatgcagataaggactc                                                                                                                                                                <  1:549257‑M1/301‑142 (MQ=255)`  
                                                                                                                                                 `ggtggtCATTGAGACCGCACAAGAAGCGATGCGCTGGGTTGGAGACCACTGGAACTTCCGGTGTCTTCTGGATACCGAAGGTAAGATGGGTCCTAATTGGGCGATTTGCCACTGATACAGGAGGCTACTCATGAACGAAAGACACTTAACAGGTGCgattgtccccaatggtaaacagaaggaaatgattgtccagttcctgacacacggtatggaaggattgatgacattcgtagtacgtacatcatttcgtgaggccattaaggacatgcacgaagagtatgcagataaggactctttc                                                                                                                                                            >  1:495506‑M1/1‑156 (MQ=255)`  
                                                                                                                                                     `gtCATTGAGACCGCACAAGAAGCGATGCGCTGGGTTGGAGACCACTGGAACTTCCGGTGTCTTCTGGATACCGAAGGTAAGATGGGTCCTAATTGGGCGATTTGCCACTGATACAGGAGGCTACTCATGAACGAAAGACACTTAACAGGTGCgattgtccccaatggtaaacagaaggaaatgattgtccagttcctgacacacggtatggaaggattgatgacattcgtagtacgtacatcatttcgtgaggccattaaggacatgcacgaagagtatgcatataaggactctttcaaac                                                                                                                                                        >  1:565102‑M1/1‑152 (MQ=255)`  
                                                                                                                                                       `cATTGAGACCGCACAAGAAGCGATGCGCTGGGTTGGAGACCACTGGAACTTCCGGTGTCTTCTGGATACCGAAGGTAAGATGGGTCCTAATTGGGCGATTTGCCACTGATACAGGAGGCTACTCATGAACGAAAGACACTTAACAGGTGCgattgtccccaatggtaaacagaaggaaatgattgtccagttcctgacacacggtatggaaggattgatgacattcgtagtacgtacatcatttcgtgaggccattaaggacatgcacgaagagtatgcagataaggactctttcaaacaa                                                                                                                                                      >  1:185920‑M1/1‑150 (MQ=255)`  
                                                                                                                                                       `cATTGAGACCGCACAAGAAGCGATGCGCTGGGTTGGAGACCACTGGAACTTCCGGTGTCTTCTGGATACCGAAGGTAAGATGGGTCCTAATTGGGCGATTTGCCACTGATACAGGAGGCTACTCATGAACGAAAGACACTTAACAGGTGCTGCTTCTGAAATGCTAGTAGCCTACAAATTTACCAAAGCTGGGTACACTGTCTATTACCCTATGCTGACTCAGAGTAAAGAGGACTTGGTTGTATGTAAGGATGGTAAATTTAGTACGGTTCAGGTTAAAAcctgtctcttatacacatct                                                                                                                                                      >  1:81149/1‑282 (MQ=255)`  
                                                                                                                                                            `agaCCGCACAAGAAGCGAGGCGCTGGGTTGGAGACCACTGGAACTGCCGGTGTCTTCTGGATACCGAAGGTAAGATGGGTCCTAATTGGGCGATTTGCCACTGATACAGGAGACTACTCATGAACTAAAGACACTTAACAGGTGCTGCTTCTGAAATGCTAGTAGCCTACAAATGTACCAAAGCTGTGTACACTGTCAATTACCCTATGCTGACTCAGAGTAAAGAGGACTTGGTTGTATGTAAGGATGGTAAATTTAGTAAGGTTCAGGTTAAAACAGCCACAACGGTTCAAACCAacac                                                                                                                                                 <  1:106366/301‑1 (MQ=255)`  
                                                                                                                                                                          `gCGATGCGCTGGGTTGGAGACCACTGGAACGTCCGGTGTCTTCTGTATACCGAAGGTAAGATGGGTCCTAATTGGGCGATTTGCCACTGATACAGGAGGCTACTCATGAACGAAAGACACTTAACAGGTGCgattgtccccaatggtaaacagaaggaaatgattgtccagttcctgacacacggtatggaaggattgatgacattcgtagtacgtacatcatttcgtgaggccattaaggacatgcacgaagagtatgcagataaggactctttcaaacaatctcctgcaacagtacggg                                                                                                                                   <  1:205500‑M1/301‑171 (MQ=255)`  
                                                                                                                                                                            `gATGCAGTGAGTTGGAGACCACGGAAAGTGACGGTGTATTCTGGATACAGAAGGTAAGATGGGTATTACTAGAGCGATTTGCCACTGATACAGGAGACTACTCATGAACAAAAGACACTTAACAGGTGCgattgtcgcgaatggtaaacagaaggaaatgattgtccagttcctgacacacgatatagaagtatagatgacattcgtagtacgtacatcatttcgtgaggccattaaggacatgcacgaagagtatgcagataaggactctttcaaacaatctcctgcaacagtacgggag                                                                                                                                 <  1:139549‑M1/301‑173 (MQ=255)`  
                                                                                                                                                                             `gagacaggggttggagaCCACTGGAACTTCCGGTGTCTTCTGGATAACGAAGGTAAGATGGGTCCTAATTGGGCGATTTGCCACTGATACAGGAGGCTACTCATGAACGAAAGACACTTAACAGGTGCgattgtccccaatggtaaacagaaggaaatgattggccagttcctgacacacggtatggaaggattgatgacattcgtagtacgtacatcatttcgtgaggccattaaggacatgcacgaagagtatgcagataaggactctttcaaacaatctcatgcaacagtacgggagg                                                                                                                                <  1:292210‑M1/294‑174 (MQ=255)`  
                                                                                                                                                                              `tGCGCTGGGTTGGAGACCACTGGAACTTCCGGTGTCTTCTGGATACCGAAGGTAAGATGGGTCCTAATTGGGCGATTTGCCACTGATACAGGAGGCTACTCATGAACGAAAGACACTTAACAGGTGCgattgtccccaatggtaaacagaaggaaatgattgtccagttcctgacacacggtatggaaggattgatgacattcgtagtacgtacatcatttcgtgaggccattaaggacatgcacgaagagtatgccgataaggactctttcaaacaatatcctgcaacagtacgggaggt                                                                                                                               >  1:100060‑M1/1‑127 (MQ=255)`  
                                                                                                                                                                                    `gggTTGGAGACCACTGGAACTTCCGGTGTCTTCTGGATACCGAAGGTAAGATGGGTCCTAATTGGGCGATTTGCCACTGATACAGGAGGCTACTCATGAACGAAAGACACTTAACAGGTGCgattgtccccaatggtaaacagaaggaaatgattgtccagttcctgacacacggtatggaaggattgatgacattcgtagtacgtacatcatttcgtgaggccattaaggacatgcacgaagagtctgcagataaggactctttcaaacaatctcctgcaacagtacgggaggtgttctg                                                                                                                         >  1:93008‑M1/1‑121 (MQ=255)`  
                                                                                                                                                                                    `gggTTGGAGACCACTGGAACTTCCGGTGTCTTCTGGATACCGAAGGTAAGATGGGTCCTAATTGGGCGATTTGCCACTGATACAGGAGGCTACTCATGAACGAAAGACACTTAACAGGTGCgatcgtccccaatggtaaacagaaggaaatgattgtccagttcctgacacacggtatggaaggattgatgacattcgtagtacgtacatcatttcgtgaggccattaaggacatgcacgaagagtatgcagataaggactctttcaaacaatctcctgcaacagtacgggaggtgttctg                                                                                                                         >  1:179016‑M1/1‑121 (MQ=255)`  
                                                                                                                                                                                    `gggTTGGAGACCACTGGAACTTCCGGTGTCTTCTGGATACCGAAGGTAAGATGGGTCCTAATTGGGCGATTTGCCACTGATACAGGAGGCTACTCATGAACGAAAGACACTTAACAGGTGCTGCTTCTGAAATGCTAGTAGCCTACAAATTTACCAAAGCTGGGTACACTGTCTATTACCCTATGCTGACTCAGAGTAAAGAGGACTTGGTTGTATGTAAGGATGGTAAATTTAGTAAGGTTCAGGTTAAAACAGCCACAACGGTTCAAACCAACTCAGGAGATGCCAAGCAGGTTAGGCt                                                                                                                         >  1:343075/1‑301 (MQ=255)`  
                                                                                                                                                                                       `ttGGAGACCACTGGAACTTCCGGTGTCTTCTGGATACCGAAGGTAAGATGGGTCCTAATTGGGCGATTTGCCACTGATACAGGAGGCTACTCATGAACGAAAGACACTTAACAGGTGCgattgtccccaatggtaaacagaaggaaatgattgtccagttcctgacacacggtatggaaggattgatgacattcgtagtacgtacatcatttcgtgaggccattaaggacatgcacgaagagtatgcagataaggactctttcaaacaatctcctgcaacagtacgggaggtgttctgatg                                                                                                                      >  1:474472‑M1/1‑118 (MQ=255)`  
                                                                                                                                                                                              `gcaCTGGAACTTCCGGTGTCTTCTGGATACCGAAGGTAAGATGGGTCCTAATTGGGCGATTTGCCAATGATACAGGAGGCTACTCATGAACGAAAGACACTTAACAGGTGCgattgtccccaatggtaaacagaaggaaatgattgtccagttcctgacacacggtatggaaggattgatgacattcgtagtacgtacatcatttcgtgaggccattaaggacatgcacgaagagtatgcagataaggactctttcaaacaatctcctgcaacagtacgggaggtgttctgatgtctgact                                                                                                               <  1:560407‑M1/300‑191 (MQ=255)`  
                                                                                                                                                                                                      `aCTTACAGTGGCTTCTGGAAACCGAAAGTAAGCTGGGTCCTAATGGGGCTATTTGCCAATGATAAAGTGTGCTACTCATGGAAGAAAGACACTTAACATGTGCTGATTCTGAAATGCTAGTAGACTACAAATTTACCAAAGCTGGGTACACTGTCTATTACCCTATGCTGACTCAGAGTAAAGAGGACTTGGTTGTATGTAAGGATGGTAAATTTAGTAAGGTTCAGGTTAAAACAGCCACAACGGTTCAAACCAACACAGGAGATGCCAAGCAGGTTAGGCTAGATGGATGCGGTAGGTc                                                                                                       <  1:479584/301‑1 (MQ=255)`  
                                                                                                                                                                                                               `gtCTTCTGGATACCGAAGGTAAGATGGGTCCTAATTGGGCGATTTGCCACTGATACAGGAGGCTACTCATGAACGAAAGACACTTAACAGGTGCgattgtccccaatggtaaacagaaggaaatgattgtccagttcctgacacacggtatggaaggattgatgacattcgtagtacgtacatcatttcgtgaggccattaaggacatgcacgaagagtatgcagataaggactctttcaaacaatctcctgcaacagtacgtgaggttttattatgtctgcctacctgacagtgctgcaa                                                                                              >  1:667944‑M1/1‑94 (MQ=255)`  
                                                                                                                                                                                                                   `tctGGATACCGAAGGTAAGATGGGTCCTAATTGGGCGATTTGCCACTGATACATGAGGCTACTCATGAACGAAAGACACTTAACAGGTGCgattgtccccaatggtaaacagaaggaaatgattgtccagttcctgacacacggtatggaaggattgatgacattcgtagtacgtacatcatttcgtgaggccattaaggacatgcacgaagagtatgcagataaggactctttcaaacaatctcctgcaacagtacgggaggtgttctgatgtctgactacctgaactgtctcttataca                                                                                          >  1:405145‑M1/1‑90 (MQ=255)`  
                                                                                                                                                                                                                          `gacagaggtaaGATGGGTCCTAATTGGGCGATTTGCCACTGATACAGGAGGCTACTCATGAACGAAAGACACTTAACAGGTGCgattgtccccaatggtaaacagaaggaaatgattgtccagttcctgacacacggtatggaaggattgatgacattcgtagtacgtacatcatttcgtgaggccattaaggacatgcacgaagagtatgcagataaggactctttcaaacaatctcctgcaacagtacgggaggtgttctgatgtctgactacctgaaagtgctgcaagcaatcaaaag                                                                                   <  1:144456‑M1/296‑219 (MQ=255)`  
                                                                                                                                                                                                                          `acagaagGTAAGATGGGTCCTAATTGGGCGATTTGCCACTGATACAGGAGGCTACTCATGAACGAAAGACACTTAACAGGTGCgattgtccccaatggtaaacagaaggaaatgattgtccagttcctgacacacggtatggaaggattgatgacattcgtagtacgtacatcatttcgtgaggccattaaggacatgcacgaagagtatgcagataaggactctttcaaacaatctcctgcaacagtacgggaggtgttctgatgtctgactacctgaaagtgctgcaagcaatcaaaag                                                                                   <  1:150730‑M1/298‑219 (MQ=255)`  
                                                                                                                                                                                                                          `aCCGAAGGTAAGATGGGTCCTAATTGGGCGATTTGCCACTGATACAGGAGGCTACTCATGAACGAAAGACAAGTAACAGGTGCgattgtccccaatggtaaacagaaggaaatgattggccagttcatgacacacggtagggaaggattgatgacattcgtagtacgtacatcatttcgtgaggccattaaggacatgcacgaagagtatgaagatgaggactctttcaaacaatctcctgcaacagtacgggaggtgttctgatgtctgactacctgaaagtgctgcaagcaatcaaaag                                                                                   <  1:123105‑M1/301‑219 (MQ=255)`  
                                                                                                                                                                                                                           `ccGAAGGTAAGATGGGTCCTAATTGGGCGATTTGCCACTGATACAGGAGGCTACTCATGAACGAAAGACACTTAACAGGTGCgattgtccccaatggtaaacagaaggaaatgattgtccagttcctgacacacggtatggaaggattgatgacattcgtagtacgtacatcatttcgtgaggccattaaggacatgtacgaggagtatgcagataaggactatttcaaacaatctcctgcaaaagtacgggaggtgttctgatgtcggactacctgaaagtgctgcaagcaataaaaatg                                                                                  >  1:621660‑M1/1‑82 (MQ=255)`  
                                                                                                                                                                                                                               `aGGTAAGATGGGTCCTAATTGGGCGATTTGCCACTGATACAGGAGGCTACTCATGAACGAAAGACACTTAACAGGTGCTGCTTCTGAAATGCTAGTAGCCTACAAATTTACCAAAGCTGGGTACACTGTCTATTACCCTATGCTGACTCAGAGTAAAGAGGACTTGGTTGTATGTAAGGATGGTAAATTTAGTAAGGTTCAGGTTAAAACAGCCACAACGGTTCAAACCAACACAGGAGATGCCAAGCAGGTTAGGCTAGGTGGATGCGGTAGGTCCGAATATAAGGATGGAGACTTTGAc                                                                              <  1:192581/301‑1 (MQ=255)`  
                                                                                                                                                                                                                                      `aggggtcCGAATTGGGCGATTTGCCACTGATACAGGAGGCTACTCATGAACGAAAGACACTTAACAGGTGCgattgtcccaaatggtaaacagaaggaaatgattgtccagttcctgacacacggtatggaaggattgatgacattcgtagtacgtacatcatttcgtgaggccattaaggacatgcacgaagagtatgcagataaggactctttcaaacaatcttctgcaacagtacgggaggtgttctgatgtctgactacctgaaagtgctgcaagcaatcaaaagttgccctaagac                                                                       <  1:44238‑M1/299‑231 (MQ=255)`  
                                                                                                                                                                                                                                        `gggTCCTAATTGGGCGATTTGCCACTGATACAGGAGGCTACTCATGAACGAAAGACACTTAACAGGTGCTGCTTCTGAAATGCTAGTAGCCTACAAATTTACCAAAGCTGGGTACACTGTCTATTACCCTATGCTGACTCAGAGTAAAGAGGACTTGGTTGTATGTAAGGATGGTAAATTTAGTAAGGTTCAGGTTAAAACAGCCACAACGGTTCAAACCAACACAGGAGATGCCAAGCAGGTTAGGCTAGGTGGATGCGGTAGGTCCGAATATAAGGATGGAGACTTTGACATTCTTGCg                                                                     >  1:370846/1‑301 (MQ=255)`  
                                                                                                                                                                                                                                           `tCCTAATTGGGCGATTTGCCACTGATACAGGAGGCTACTCATGAACGAAAGACACTTAACAGGTGCtcttgtccccaatggtaaacagacggaaatgattgtccagttcctgacacacggtattgaaggattgatgacattcttattacgtacatcatttcgtgaggccattaaggacatgcacgaagcgtatgcagataaggactctttcaaacaatctcctgcaacagtacgggaggtgttctgatgtctgactacctgaaagtgctgcaagcaatcacaagttgccctaagactttcc                                                                  >  1:660906‑M1/1‑66 (MQ=255)`  
                                                                                                                                                                                                                                           `ggagatgtgtaaaagagacagctgatacaggaggctactcatgAACGAAAGACACTTAACAGGTGCgattgtccccaatggtaaacagaaggaaatgattgtccagttcctgacacacggtatggaaggattgatgacattcgtagtacgtacatcatttcgtgaggccattaaggacatgcacgaagagtatgcagataaggactctttcaaacaatctcctgcaacagtacgggaggtgttctgatgtctgactacctgaaagtgctgcaagcaatcaaaagttgccctaagactttcc                                                                  <  1:490660‑M1/280‑236 (MQ=255)`  
                                                                                                                                                                                                                                                        `agagacagctgatacagGAGGCTACTCATGAACGAAAGACACTTAACAGGGGCgattgtccccaatggtaaacagaaggaaatgattgtccagttcctgacacacggtatggaaggattgatgacattcgtagtacgtacatcatttcgtgaggccattaaggacatgcacgaagagtatgcagataaggactctttcaaacaatctcctgcaacagtacgggaggtgttctgatgtctgactacctgaaagtgctgcaagcaatcaaaagttgccctaagactttccagtccaactatgt                                                     <  1:609105‑M1/293‑249 (MQ=255)`  
                                                                                                                                                                                                                                                               `aCTGATACAGGAGGCTACTCATGAACGAAAGACACTTAACAGGTGCgattgtccccaatggtaaacagaaggaaatgattgtccagttcctgacacacggtatggaaggattgatgacattcgtagtacgtacatcatttcgtgaggccattaaggacatgcacgaagagtatgcagataaggactctttcaaacaatctcctgcaacagtacgggaggtgttctgatgtctgactacctgaaagtgctgcaagcaatcaaaagttgccctaagactttccagtccaactatgtacggaac                                              <  1:21919‑M1/301‑256 (MQ=255)`  
                                                                                                                                                                                                                                                                `cTGATACAGGAGGCTACTCATGAACGAAAGACACTTAACAGGTGCTGCTTCTGAAATGCTAGTAGCCTAAAAATTTACCAAAGCTGGGTACACTGTCTATTACCCTATGCTGACTCAGAGTAAAGAGGACTTGGTTGTATGTAAGGATGGTAAATTTAGTAAGGCTCAGGTTAAAACAGCCACAACGGTTCAAACCAACACAGGAGATGCCAAGCAGGTTAGGCTAGGTGGATGCGGCGGGTCCGAATATAAGGATGGAGACTTTGACATTCTTGCGGTTGTGGTTGACGAAGATGTGCtt                                             >  1:501609/1‑301 (MQ=255)`  
                                                                                                                                                                                                                                                                 `tGATACAGGAGGCTACTCATGAACGAAAGACACTTAACAGGTGCTGCTTCTGAAATGCTAGTAGCCTACAAATTTACCAAAGCTGGGTACACTGTCTATTACCCTATGCTGACTCAGAGTAAAGAGGACTTGGTTGTATGTAAGGATGGTAAATTTAGTAAGGTTCAGGTTAAAACAGCCACAACGGTTCAAACCAACACAGGAGATGCCAAGCAGGTTAGGCTAGGTGGATGCGGTAGGTCCGAATATAAGGATGGAGACTTTGACATTCTTGCGGTTGTGGTTGACGAAGATGTGCttt                                            >  1:232393/1‑300 (MQ=255)`  
                                                                                                                                                                                                                                                                 `tGATACAGGAGGCTACTCATGAACGAAAGACACTTAACAGGTGCTGCTTCTGAAATGCTAGTAGCCTACAAATTTACCAAAGCTGGGTACACTGTCTATTACCCTATGCTGACTCAGAGTAAAGAGGACTTGGTTGTATGTAAGGATGGTAAATTTAGTAAGGTTCAGGTTAAAACAGCCACAACGGTTCAAACCAACACAGGAGATGCCAAGCAGGTTAGGCTAGGTGGATGCGGTAGGTCCGAATATAAGGATGGAGACTTTGACATTCTTGCGGTTGTGGTTGACGAAGATGTGCTTa                                            >  1:615328/1‑301 (MQ=255)`  
                                                                                                                                                                                                                                                                    `aacAGGAGGCTACTCATGAACGAAAGACACTTAACAGGTGCgattgtccccaatggtaaacagaaggaaatgattgtccagttcctgacacacggtatggaaggattgatgacattcgtagtacgtacatcatttcgtgaggccattaaggacatgcacgaagagtatgcagataaggactctttcaaacaatctcctgcaacagtacgggaggtgttctgatgtctgactacctgaaagtgctgcaagcaatcaaaagttgccctaagactttccagtccaactatgtacggaacaatgc                                         <  1:526127‑M1/300‑261 (MQ=255)`  
                                                                                                                                                                                                                                                                         `gaggCTTCTCATGAACGAAAGACCCTTAACAGGTGCgattgtccccaatggtaaacagaaggaaattagtgtccagttcctgacacacggtatggaaggattgatgacattcgtagtacgtacatcatttcgtgaggccattaaggacatgcacgaagagtatgcagataaggactctttcaaacaatctcctgcaacagtacgggaggtgttctgatgtctgactacctgaaagtgctgcaagcaatcaaaagttgccctaagactttccagtccaactatgtacggaacaatgcgagcc                                    <  1:260283‑M1/301‑266 (MQ=255)`  
                                                                                                                                                                                                                                                                                `cTCATGAACGAAAGACACTTAACAGGTGCTGCTTCTGAAATGCTAGTAGCCTACAAATTTACCAAAGCTGGGTACACTGTCTATTACCCTATGCTGACTCAGAGTAAAGAGGACTTGGTTGTATGTAAGGATGGTAAATTTAGTAAGGTTCAGGTTAAAACAGCCACAACGGTTCAAACCAACACAGGAGATGCCAAGCAGGTTAGGCTAGGTGGATGCGGTAGGTCCGAATATACGGATGGAGACTTTGACATTCTTGCGGTTGTGGTTGACGAAGATGTGCTTATTTTCACATGGGACg                             >  1:604338/1‑301 (MQ=255)`  
                                                                                                                                                                                                                                                                                  `cATGAACGAAAGACACTTAACAGGTGCgattgtccccaatggtaaacagaaggaaatgattgtccagttcctgacacacggtatggaaggattgatgacattcgtagtacgtacatcatttcgtgaggccattaaggacatgcacgaagagtatgcagataaggactctttcaaacaatctcctgcaacagtacgggaggtgttctgatgtctgactacctgaaagtgctgcaagcaatcaaaagttgccctaagactttccagtccaactatgtacggaacgatgcgagcctcgtagcgg                           <  1:431209‑M1/301‑275 (MQ=255)`  
                                                                                                                                                                                                                                                                                  `cATGAACGAAAGACACTTAACAGGTGCgattgtccccaatggtaaacagaaggaaatgattgtccagttcctgacacacggtatggaaggattgatgacattcgtagtacgtacatcatttcgtgaggccattaaggacatgcacgaagagtatgcagataaggactctttcaaacaatctcctgcaacagtacgggaggtgttctgatgtctgactacctgaaagtgctgcaagcaatcaaaagttgccctaagactttccagtccaactatgtacggaacaatgcgagcctcgtagcgg                           <  1:15679‑M1/301‑275 (MQ=255)`  
                                                                                                                                                                                                                                                                                  `cATGAACGAAAGACACTTAACAGGTGCgattgtccccaatggtaaacagaaggaaatgattgtccagttactgacacactgtatggaaggattgatgacattcgtagtacgtacatcatttcgtgaggccattaaggacatgcacgaagagtatgcagataaggactctttcaaacaatctcctgcaacagtacgggaggtgttctgatgtctgactacctgaaagtgctgcaagcaatcaaaagttgccctaagactttccagtccaactatgtacggaacaatgcgagcctcgtagcgg                           <  1:454922‑M1/301‑275 (MQ=255)`  
                                                                                                                                                                                                                                                                                      `aaCGAAAGACACTTAACAGGTGCgattgtccccaatggtaaacagaaggaaatgattgtccagttcctgacacacggtatggaaggattgatgacattcgtagtacgtacatcatttcgtgaggccattaaggacatgcacgaagagtatgcagataaggactctttcaaacaatctcctgcaacagtacgggaggtgttctgatgtctgactacctgaaagtgctgcaagcaatcaaaagttgccctaagactttccagtccaactatgtacggaacaatgcgagcctcgtagcggaggc                       >  1:12766‑M1/1‑23 (MQ=255)`  
                                                                                                                                                                                                                                                                                                 `cTTAACAGGTGCgattgtccccaatggtaaacagaaggaaatgattgtccaggtcctgacacacggtatggaaggattgatgacattcgtagtacgtacatcatttcgtgaggccattaaggacatgcacgaagagtatgcagataaggacactttcaaacaatatcctgcaacagtacgggcggtgttctgatgtctgactacctgaaagtgctgcaagcaatcaaaagttgccctaagactttccattccaactatgtacggaacaatgcgagcctcgtagaggaggccgcttcctgtt            >  1:55399‑M1/1‑12 (MQ=255)`  
                                                                                                                                                                                                                                                                                                 `cTTAACAGGTGCTGCTTCTGAAATGCTAGTAGCCTACAAATTTACCAAAGCTGGGTACACTGTCTATTACCCTATGCTGACTCAGCGTAAAGAGGACTTGGTTGTATGTAAGGATGGTAAATTTAGTAAGGTTCAGGTTAAAACAGCCACAACGGTTCAAACTAACACAGGAGATGCCAAGCAGGTTAGGCTAGGTGGATGCGGTAGGTCCGAATATAAGGATGGAGACTTTGACATTCTTGCGGTTGTGGTTGACGAAGATGTGCTTATTTTCACATGGCACGAAGTAAAAGGTAAGAcc            >  1:647713/1‑300 (MQ=255)`  
                                                                                                                                                                                                                                                                                                 `cTTAACAGGTGCTGCTTCTGAAATGCTAGTAGCCTACAAATTTACCAAAGCTGGGTACACTGTCTATTACCCTATGCTGACTCAGAGTAAAGAGGACTTGGTTGTATGTAAGGATGGTAAATTTAGTAAGGTTCAGGTTAAAACAGCCACAACGGTTCAAACCAACACAGGAGATGCCAAGCAGGTTAGGCTAGGTGGATGCGGTAGGTCCGAATATAAGGATGGAGACTTTGACATTCTTGCGGTTGTGGTTGACGAAGATGTGCTTATTTTCACATGGGACGAAGTAAAAGGTAAGACa            >  1:113055/1‑301 (MQ=255)`  
                                                                                                                                                                                                                                                                                                   `tAACAGGTGCgattgtccccaatggtaaacagaaggaaatgattgtccagttcctgacacacggtatggaaggattgatgacattcgtagtacgtacatcatttcgtgaggccattaaggacatgcacgaagagtatgcagataaggactctttcaaacaatctcctgcaacagtacgggaggtgttctgatgtctgactacctgaaagtgctgcaagcaatcaaaagttgccctaagactttccagtccaactatgtacggaacaatgcgagcctcgtagcggaggccgcttcccgtggt          >  1:484602‑M1/1‑10 (MQ=255)`  

                                                                                                                                                                                                                                                                                                            `|`                                                                                                                                                                                                                                                                                                             
`CTCTCATCTGCAAACTGTGGATTATCAAGACCGAAGAGATGCTCGTAGAGAAAGGCTTGAAGCATGGCTGGGATGGGGACTTTGCGTACATGGCATGGGTACATGATGAAATCCAAGTAGGCTGCCGTACCGAAGAGATTGCTCAGGTGGTCATTGAGACCGCACAAGAAGCGATGCGCTGGGTTGGAGACCACTGGAACTTCCGGTGTCTTCTGGATACCGAAGGTAAGATGGGTCCTAATTGGGCGATTTGCCACTGATACAGGAGGCTACTCATGAACGAAAGACACTTAACAGGTGCTGCTTCTGAAATGCTAGTAGCCTACAAATTTACCAAAGCTGGGTACACTGTCTATTACCCTATGCTGACTCAGAGTAAAGAGGACTTGGTTGTATGTAAGGATGGTAAATTTAGTAAGGTTCAGGTTAAAACAGCCACAACGGTTCAAACCAACACAGGAGATGCCAAGCAGGTTAGGCTAGGTGGATGCGGTAGGTCCGAATATAAGGATGGAGACTTTGACATTCTTGCGGTTGTGGTTGACGAAGATGTGCTTATTTTCACATGGGACGAAGTAAAAGGTAAGACATCCATGTGTG  >  NC_001604.1/16208‑16807`

  

Alignment Legend

Aligned base mismatch/match (shaded by quality score): `ATCG/ATCG` `< 3 ≤` `ATCG/ATCG` `< 10 ≤` `ATCG/ATCG` `< 22 ≤` `ATCG/ATCG` `< 34 ≤` `ATCG/ATCG` `< 38 ≤` `ATCG/ATCG`

Unaligned base: `atcg`    Masked matching base: `atcg`    Alignment gap: `‑`    Deleted base: `‑`

Reads not counted as support for junction

read\_name Not counted due to insufficient overlap past the breakpoint.

read\_name Not counted due to not crossing MOB target site duplication.
